# Tailoring Antimicrobial Stewardship (AMS) Interventions to the Cultural Context: An Investigation of AMS Programs Operating in Northern Italian Acute-Care Hospitals

**DOI:** 10.3390/antibiotics11091257

**Published:** 2022-09-16

**Authors:** Costanza Vicentini, Valentina Blengini, Giulia Libero, Manuela Martella, Carla Maria Zotti

**Affiliations:** Department of Public Health and Pediatrics, University of Turin, Via Santena 5 bis, 10126 Turin, Italy

**Keywords:** antimicrobial resistance, antimicrobial stewardship, Italy, implementation science

## Abstract

Antibiotic misuse and overuse are important contributors to the development of antimicrobial resistance (AMR). Antimicrobial stewardship (AMS) programs are coordinated sets of actions aiming to promote appropriate antibiotic use, improving patient outcomes whilst reducing AMR. Two main organizational models for AMS programs have been described: restrictive strategies (RS) vs. enabling strategies (ES). Evaluating and understanding social and cultural influences on antibiotic decision-making are critical for the development of successful and sustainable context-specific AMS programs. Characteristics and surrogate outcomes of AMS programs operating in acute-care hospitals of Piedmont in north-western Italy were investigated. The aim of this study was assessing whether RS vs. ES operating in our context were associated with different outcomes in terms of total antimicrobial usage and percentage of methicillin-resistant *Staphylococcus aureus* (MRSA) and carbapenem-resistant enterobacteria (CRE) over invasive isolates. In total, 24 AMS programs were assessed. ES were more frequently chosen compared to RS, with the latter being implemented only in broader AMS programs involving enabling components (combined strategy, CS). This study found no difference in evaluated outcomes among hospitals implementing ES vs. CS, suggesting both approaches could be equally valid in our context.

## 1. Introduction

Antimicrobial resistance (AMR) is recognized as a global public health threat. Antibiotic misuse and overuse are important contributors to the development of AMR, due to the ecological impact of these agents. National and international initiatives to promote the judicious use of antibiotics have been developed to preserve the effectiveness of these agents [1]. Antimicrobial stewardship (AMS) programs are coordinated sets of actions aiming to promote appropriate antibiotic use, improving patient outcomes whilst reducing AMR [2,3]. Two main organizational models for AMS programs have been described: interventions based on pre-prescription authorization (restrictive or front-end strategies) vs. post-prescription review and feedback (enabling or back-end strategies) [4]. The 2016 Infectious Disease Society of America (IDSA) and Society for Healthcare Epidemiology of America (SHEA) guidelines for implementing AMS programs strongly recommended both approaches [4]. More recently, a Cochrane review summarized evidence from over 200 studies on the safety and effectiveness of interventions aiming to ameliorate antimicrobial prescribing in acute-care settings. Results of the study indicated that both enablement and restriction were associated with greater intervention effect (enablement: beta at meta-regression of randomized controlled trials, RCTs, 15.12, 95% confidence interval, CI, 8.45 to 21.8; restriction: beta at meta-regression of RCTs 34.91, 95% CI 13.52–56.29), and that interventions that included feedback were more effective compared to those that did not include feedback (beta at meta-regression of RCTs 10.88, 95% CI 7.16–19.32). The Authors concluded that enabling strategies consistently increased the impact of interventions, including interventions with a restrictive component [5].

A growing body of the literature has been dedicated to behavior change and implementations strategies, investigating *why* AMS interventions are or are not effective [6]. The impact of any quality improvement initiative in healthcare depends heavily on setting-specific social, psychological, organizational, and cultural dynamics. Context in particular has been identified as a major challenge for quality improvement efforts [7]. Historically, AMS interventions have been implemented without factoring in these elements, despite several studies highlighting the importance of tailoring interventions to local contexts in order for them to be impactful and sustainable [8]. The decision on whether to prescribe an antibiotic is a complex process, influenced by several factors other than physicians’ attitudes and beliefs, such as social and cultural norms [9,10]. Even though a number of studies have provided insight into the impact of behavioral and social influences on antimicrobial prescribing practices in different settings [9], cultural, organizational and interpersonal determinants of antibiotic decision-making remain under-explored [1,6].

Adapting both interventions and implementation strategies to fit with context-specific cultures, practices, and care systems is recognized as key to the success of quality improvement efforts. Interventions should be aligned with local barriers and opportunities, and should reflect the priorities of stakeholders at all levels, from practitioners to the wider system-level [7]. Other than challenges pertaining to resources, infrastructure, case-mix, and healthcare-associated infections rates, several cultural and context-specific determinants of AMS have been identified [1,9]. A recent qualitative study found governmental involvement had very different impacts on AMS in high-income (HICs) vs. lower- to middle-income countries (LMICs). Conversely, local championing and leadership was identified as a significant facilitator, irrespective of income and governmental involvement [11]. In their global survey, Nampoothiri et al. identified universal behaviors associated with antibiotic decision-making, which were less linked to countries’ income status and more related to cultural and contextual practices [9]. Evaluating and understanding social and cultural influences on antibiotic decision-making are critical for the development of successful and sustainable AMS programs [6,11].

A recent study by Shallal et al. published in *Antibiotics* evaluated the impact of a post-prescription review and feedback AMS program operating in a tertiary-care hospital in Lebanon [12]. The authors found the intervention engaged physicians in discussions, provided a platform for education, and fostered collaborative decision-making concerning antibiotic prescription. Interdisciplinary and multifaceted approaches have been identified as important elements for AMS intervention success [9,13]. Furthermore, the study by Shallal et al. included a survey of physicians’ attitudes towards the program, which revealed a high level of acceptability of the program (88%) and interesting cultural considerations, including the importance of continuous education, of placing infectious disease specialists rather than pharmacists in AMS leadership roles, and of locally developed guidelines [12]. A previous systematic review of studies conducted in the Middle East highlighted the impact of cultural elements such as physician attitudes and acceptance of collaborative practices on the effectiveness of AMS interventions [14].

AMS interventions are particularly challenging in Italy. Italy is among the highest consumers of antibiotics in general, and of broad-spectrum agents in particular, in Europe [15,16]. Italian AMR rates for several pathogens are considered hyper-endemic [17]. The 2017 European Center for Disease Prevention and Control (ECDC) country visit to discuss AMR found the high AMR rates in Italy appeared to be accepted and considered unavoidable by all stakeholders, with little sense of urgency, institutional support, professional leadership, accountability, and coordination of activities at all levels. According to the report, the regional framework of healthcare provision in Italy hinders the achievement of cohesive and standardized action nationwide. A comprehensive, centrally coordinated response is required, and local/regional experiences of good practices should be shared and expanded across the country [17].

Reducing AMR rates and improving antibiotic prescribing practices, including through AMS programs, are recognized as urgent priorities in our country [18]. AMS interventions are implemented in the majority of Italian acute-care hospitals. However, AMS programs are not mandatory, and the effectiveness of single interventions as well as of broader AMS programs implemented in Italy remains to be determined.

## 2. Results

In the region of Piedmont, in North-western Italy, characteristics and surrogate outcomes of AMS programs operating in acute-care hospitals are routinely monitored, as part of the regional healthcare-associated infections (HAI) and AMR prevention and control program. Our previous analysis of data reported through the regional program found AMS interventions were implemented in all trusts, albeit with important inter-facility differences. Improvements in several outcome metrics were found: total antimicrobial usage decreased by 4% between 2017 and 2019, while AMR rates decreased by 16% and 23%, respectively, for the percentage of methicillin-resistant *Staphylococcus aureus* (MRSA) and carbapenem-resistant enterobacteria (CRE) over invasive isolates [19]. According to our survey, enabling strategies were implemented more frequently compared to restrictive strategies, however we did not investigate the impact of choice of strategy on outcome metrics in our previous publication [19].

Results of several studies suggest post-prescription review and feedback AMS programs are effective in both HICs and LMICs [5,12], and could be more acceptable than restrictive strategies [20]. For the purpose of this study, we conducted further analyses on data collected through our previous study, with the objective of determining if enabling and restrictive AMS strategies operating in our context were associated with different outcomes in terms total antimicrobial usage, MRSA and CRE rates.

The methodology for data collection and applied definitions were described at length in our previous publication [19]. Briefly, AMS programs implemented in public and private trusts of Piedmont were investigated through a survey part of the regional HAI and AMR prevention and control program. Data on 2017–2019 were collected. Hospital characteristics such as ownership, level of care (secondary, tertiary, teaching and specialized), number of beds, and number of full time equivalent (FTE) dedicated infection control personnel per 100 beds were recorded. The survey also included open questions investigating characteristics and elements of AMS programs. Concerning outcome indicators, the annual means and percentage change between the years 2017–2019 were calculated. Total antimicrobial usage was expressed in defined daily doses (DDD) per 1000 patient-days (pds).

For the purpose of this study, based on survey responses AMS strategies were classified as restrictive (RS), enabling (ES), or combined (restrictive and enabling, CS). Strategies were classified as RS if they included pre-prescription authorization, i.e., if physicians required the authorization of an infectious disease (ID) consultant in order to be able to prescribe any antibiotic or a specific antimicrobial agent, most often broad-spectrum or novel agents. Strategies were classified as ES if they were based on ID consultants performing post-prescription reviews of some or all prescribed antibiotic agents, performing audits, and providing feedback to prescribers [20]. If both elements were included, the strategy was classified as CS. Differences in characteristics and outcome indicators among AMS strategy groups were investigated using Kruskal–Wallis tests. Univariate analysis using a generalized linear model (GLM) was run to assess the association between outcomes and hospital characteristics (size, ownership, level of care), as well as AMS strategy. All analyses were conducted using SPSS v. 27.0 (SPSS Inc., Armonk, NY, USA), with two-tailed statistical significance set at <0.05.

No ethical approval was sought for the current study, as it was part of a quality improvement initiative coordinated by a public entity (Region of Piedmont), and considering no patient-level data were collected.

## 3. Discussion

**Figure 1:**
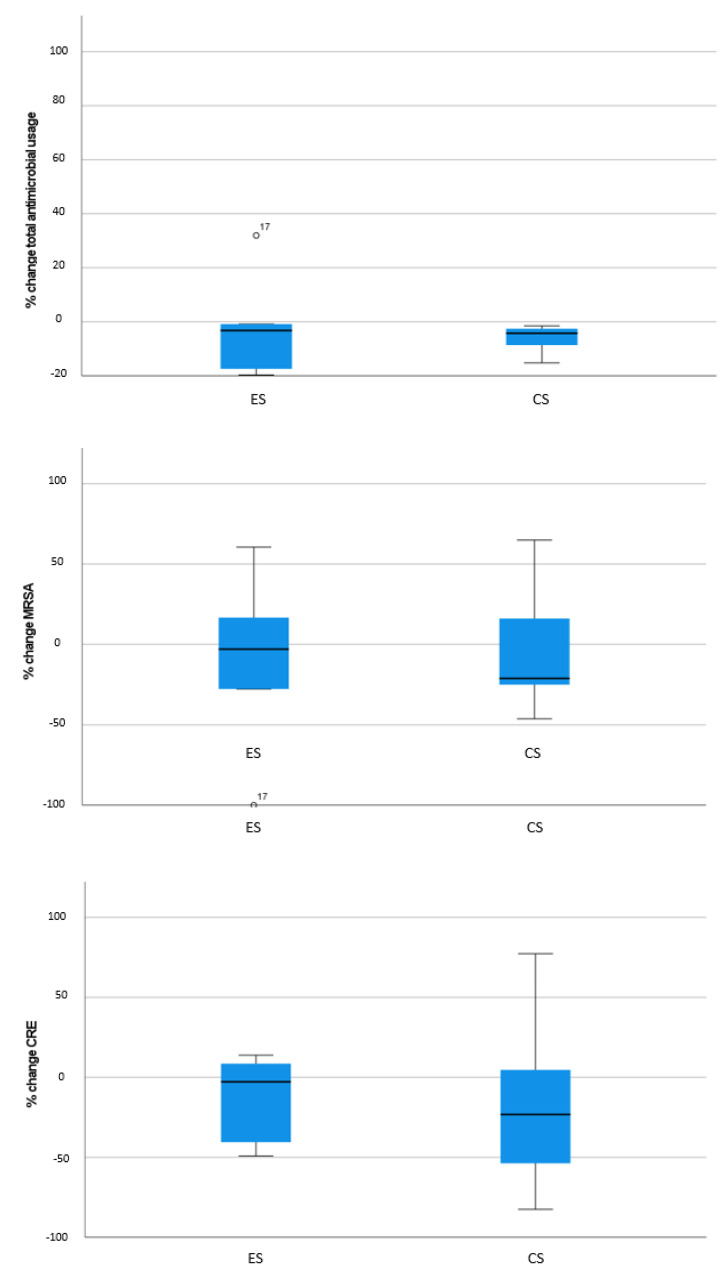
Percentage change in antimicrobial stewardship (AMS) outcome metrics of acute-care hospitals of the region of Piedmont, Italy, 2017–2019, stratified by AMS strategy: enabling (ES, *n* = 7) vs. combined enabling and restrictive (CS, *n* = 17).

In total, 24 AMS programs were assessed: 19 operating in public and 5 in private hospitals. Data on AMR was available from all 24 hospitals, whereas data on antimicrobial usage was obtained from 19 hospitals. The majority of hospitals implemented CS (*n* = 17), 7 implemented ES, and no hospital implemented RS alone. CS included the following elements: pre-prescription authorization (*n* = 17), auditing (*n* = 15), developing local guidelines (*n* = 3) and diagnostic stewardship interventions (*n* = 2). Characteristics of participating hospitals and considered outcomes according to AMS strategy (ES vs. CS) are summarized in Table 1. As shown in Figure 1, no significant difference among hospitals stratified according to AMS strategy was identified for any of the considered outcomes.

Univariate analysis also failed to identify significant changes in outcome measures according to AMS strategy. As shown in Table 2, the only characteristic significantly associated with the considered outcomes was level of care: significantly higher changes in antimicrobial usage and CRE were identified in hospitals providing secondary compared to tertiary care.

## 4. Conclusions

This study had several limitations. The generalizability of results of this study could be limited by selection and self-reporting biases. Even though the majority of hospitals in the region participated in the survey, the number of observations was relatively small as programs were evaluated at the hospital level. Three hospitals were excluded from analyses on antimicrobial use, as they did not provide consumption data. Further, we cannot exclude other unmeasured factors could have led to changes in outcome indicators, such as temporal trends, seasonality, and differences in antibiotics classes included in AMS interventions, as well as the presence of a dedicated infection control team.

Despite its limitations, our study investigated differences in AMS strategies implemented by 24 acute-care hospitals of Northern Italy. ES were more frequently chosen compared to RS, with the latter being implemented only in broader AMS programs involving enabling components. This study found no difference in change in antimicrobial usage and AMR rates among hospitals implementing ES vs. CS, suggesting both approaches could be equally valid in our context. Further research should focus on understanding local determinants of antibiotic prescribing, as well as barriers and facilitators to AMS, in order to improve the design and implementation of contextually fit AMS programs.

## Figures and Tables

**Table 1:** Characteristics and antimicrobial stewardship (AMS) outcome metrics stratified by AMS strategy of acute-care hospitals of the region of Piedmont, Italy, 2017–2019 (N = 24).

	Hospitals Implementing Enabling AMS Strategies (ES), *n* = 7	Hospitals Implementing Combined Enabling and Restrictive AMS Strategies (CS), *n* = 17
**Characteristics**		
Ownership, N (%)		
Public	4 (57.14)	15 (88.24)
Private	3 (42.86)	2 (11.76)
Level of care, N (%)		
Secondary	3 (42.86)	6 (35.29)
Tertiary	1 (14.29)	8 (47.06)
Teaching	0	3 (17.65)
Specialized	3 (42.86)	0
N of beds, median (IQR)	333 (135–432)	526 (247.5–624.5)
Number of dedicated FTE infection control nurses per 100 beds, median (IQR)	0.46 (0.3–0.58)	0.53 (0.41–0.67)
**Outcomes**		
Total antimicrobial usage, median (IQR) % change in DDD per 1000 pds	−3.23 (−18.54–15.57)	−4.21 (−9.89–−2.44)
MRSA, median (IQR) % change	−2.96 (−45.87–27.63)	−21.19 (−25.5–21.02)
CRE, median (IQR) % change	−2.77 (−44.8–11.21)	−23.23 (−57.43–10.35)

CRE: proportion of carbapenem resistance among Acinetobacter spp., *Escherichia coli*, *Pseudomonas aeruginosa* and *Klebsiella pneumoniae* invasive isolates; DDD: defined daily doses; FTE: full time equivalent; IQR: inter-quartile range; MRSA: proportion of oxacillin and cefoxitin resistance among *S. aureus* invasive isolates.

**Table 2:** Univariate analysis of hospital characteristics and antimicrobial stewardship (AMS) strategy in relation to outcome metrics, Piedmont, Italy, 2017–2019 (*n* = 24).

	Percentage Change in Antimicrobial Usage		Percentage Change in MRSA		Percentage Change in CRE	
	Coefficient (95% CI)	*p* Value	Coefficient (95% CI)	*p* Value	Coefficient (95% CI)	*p* Value
Hospital size						
>400 beds	Ref		Ref		Ref	
200–400 beds	−1.84 (−12.22–8.53)	0.728	23.9 (−10.84–58.64)	0.177	22.26 (−15.69–60.21)	0.250
<200 beds	9.55 (−2.98–22.08)	0.135	−7.7 (−45.44–30.05)	0.689	27.12 (−22.14–76.37)	0.281
Level of care						
Tertiary	Ref		Ref		Ref	
Secondary	52.31 (0.38–104.24)	**0.048**	6.82 (−25.09–38.74)	0.675	38.23 (4.7–71.75)	**0.025**
Teaching	9.03 (−61–79.05)	0.177	10.44 (−33.34–54.23)	0.64	2.23 (−45.18–49.64)	0.259
Specialized	−63.75 (−156.38–28.89)	0.801	−33.95 (−85.3–17.39)	0.195	43.13 (−31.83–118.1)	0.926
Ownership						
Private	Ref		Ref		Ref	
Public	43.99 (−34.07–122.05)	0.269	14.33 (−23.64–52.3)	0.459	−20.09 (−69.27–29.1)	0.423
AMS strategy						
Enabling (ES)	Ref		Ref		Ref	
Combined (CS)	−48.96 (−113.46–15.54)	0.137	−3.48 (−36.74–29.78)	0.837	3.5 (−37.33–44.33)	0.867

## Data Availability

Data will be made available upon reasonable request.
